# Tissue-specific DNA methylation variability and its potential clinical value

**DOI:** 10.3389/fgene.2023.1125967

**Published:** 2023-07-19

**Authors:** Ryan H. Miller, Chad A. Pollard, Kristin R. Brogaard, Andrew C. Olson, Ryan C. Barney, Larry I. Lipshultz, Erica B. Johnstone, Yetunde O. Ibrahim, James M. Hotaling, Enrique F. Schisterman, Sunni L. Mumford, Kenneth I. Aston, Tim G. Jenkins

**Affiliations:** ^1^ Inherent Biosciences, Salt Lake City, UT, United States; ^2^ Department of Cell Biology and Physiology, Brigham Young University, Provo, UT, United States; ^3^Scott Department of Urology, Baylor College of Medicine, Houston, TX, United States; ^4^Division of Reproductive Endocrinology and Infertility, Department of Obstetrics and Gynecology, University of Utah School of Medicine, Salt Lake City, UT, United States; ^5^Division of Reproductive Endocrinology and Infertility, Department of Obstetrics and Gynecology, University of Texas Health Science Center in San Antonio, San Antonio, TX, United States; ^6^Division of Urology, Department of Surgery, University of Utah School of Medicine, Salt Lake City, UT, United States; ^7^Department of Biostatistics, Perelman School of Medicine, University of Pennsylvania, Philadelphia, PA, United States; ^8^ Epidemiology Branch, Division of Intramural Population Health Research, Eunice Kennedy Shriver National Institute of Child Health and Human Development, Bethesda, MD, United States

**Keywords:** DNA methylation, gene promoter methylation, gene promoter dysregulation, sperm DNA, male infertility

## Abstract

Complex diseases have multifactorial etiologies making actionable diagnostic biomarkers difficult to identify. Diagnostic research must expand beyond single or a handful of genetic or epigenetic targets for complex disease and explore a broader system of biological pathways. With the objective to develop a diagnostic tool designed to analyze a comprehensive network of epigenetic profiles in complex diseases, we used publicly available DNA methylation data from over 2,400 samples representing 20 cell types and various diseases. This tool, rather than detecting differentially methylated regions at specific genes, measures the intra-individual methylation variability within gene promoters to identify global shifts away from healthy regulatory states. To assess this new approach, we explored three distinct questions: 1) Are profiles of epigenetic variability tissue-specific? 2) Do diseased tissues exhibit altered epigenetic variability compared to normal tissue? 3) Can epigenetic variability be detected in complex disease? Unsupervised clustering established that global epigenetic variability in promoter regions is tissue-specific and promoter regions that are the most epigenetically stable in a specific tissue are associated with genes known to be essential for its function. Furthermore, analysis of epigenetic variability in these most stable regions distinguishes between diseased and normal tissue in multiple complex diseases. Finally, we demonstrate the clinical utility of this new tool in the assessment of a multifactorial condition, male infertility. We show that epigenetic variability in purified sperm is correlated with live birth outcomes in couples undergoing intrauterine insemination (IUI), a common fertility procedure. Men with the least epigenetically variable promoters were almost twice as likely to father a child than men with the greatest number of epigenetically variable promoters. Interestingly, no such difference was identified in men undergoing *in vitro* fertilization (IVF), another common fertility procedure, suggesting this as a treatment to overcome higher levels of epigenetic variability when trying to conceive.

## Introduction

In 2003, one of the most profound efforts ever undertaken in the biological sciences, the Human Genome Project, was completed (Collins et al., 2003). At the time there was a great deal of hope that unlocking the genetic code was the key to diagnosing and treating the vast majority of diseases. While the discoveries made have been of great interest to many and have opened the door for important genetic and epigenetic findings, clinically meaningful impacts remain elusive for many diseases. In large part, this is due to the complex, multifactorial nature of most disease processes, with etiologies resulting from a constellation of genetic, epigenetic, and environmental perturbations.

In hindsight, the lack of clinical efficacy for both genetic and epigenetic findings is not surprising. While it is common to identify a genetic or epigenetic association to a given disease, those associations occur so rarely in a population, or are only one of many alterations required to generate a pathological phenotype, that it is unreasonable to produce a highly predictive screening test capable of impacting clinical care. As a result, better approaches for analysis of genetic and epigenetic data are needed to identify clinically actionable predictors of disease or disease progression. Molecular diagnostic research and technologies need to focus, not on single genes or independent epigenetic modifications associated with a pathology, but on a comprehensive screen of alterations to gene regulatory activity at a myriad of critical genes.

To address this need, we present a novel and simple analytic method that allows for the molecular (epigenetic) assessment of complex diseases. As opposed to traditional methylation analyses that detect differentially methylated regions, this approach analyzes multi-pathway epigenetic regulation and is based on the concept that there is stable DNA methylation variability at gene promoters in healthy tissue types. Here we outline the development and utility of this new method in predicting tissue-specific disease states through the analysis of DNA methylation array data from over 2,400 samples and 20 cell types. This work first validates that promoter methylation variability is tissue-specific, and shows that the most stable gene promoters are associated with genes critical for specific tissue function. Second, it establishes that by only looking at promoter methylation variability we can distinguish between healthy and diseased tissues. Finally, by looking at a single specific tissue type (sperm), this method demonstrates direct clinical utility by showing men with high gene-promoter methylation variability in their sperm have low pregnancy and live birth rates.

## Materials and methods

### Data collection

Several publicly available datasets were used in this study. Infinium HumanMethylation450 Bead Chip data was obtained for tumor and healthy tissue samples (*n* = 494) from The Cancer Genome Atlas (TCGA) Program as compiled by the University of California Santa Cruz Xena Functional Genomics Explorer (Goldman et al., 2020) (https://xenabrowser.net/datapages/). Infinium HumanMethylation450 Bead Chip data for CD4^+^ T cell (*n* = 11), CD8^+^ T cell (*n* = 14), Alzheimer’s disease and control brain (*n* = 190), lung (*n* = 6), liver (*n* = 26), and skin methylation data (*n* = 18) from healthy and diseased individuals were accessed from the NIH Gene Expression Omnibus (GSE130029, GSE130030, GSE66351, GSE51077, GSE61258, GSE115797, respectively).

Sperm Infinium HumanMethylation450 Bead Chip data from fertile sperm donors as well as patients undergoing *in vitro* fertilization (IVF) (*n* = 166) was used from a previously published single-site study by Aston, et al. (Aston et al., 2015) as well the sperm Infinium MethylationEPIC Array data from a clinical multi-site study of patients being seen by physicians for fertility care (*n* = 1,287) as published by Jenkins et al. (Jenkins et al., 2021).

It is important to note that data in this study were only ever analyzed, visualized, and compared to data run on the sample microarray platform; i.e., samples run on the Infinium HumanMethylation450 Bead Chip were only compared to and visualized with other samples run on the Infinium HumanMethylation450 Bead Chip.

### Sample collection

Semen samples were procured from the University of Utah Andrology department from consented patients undergoing fertility care (*n* = 63), as well as two independent fertile sperm donor cohorts (*n* = 64). Semen samples from consented patients seeking fertility care were also procured from the Urology Department at Baylor College of Medicine (*n* = 49).

### Sample preparation

For all semen samples, somatic cell lysis, sperm isolation, DNA extraction, and bisulfite conversion were performed as described by Aston, et al. (Aston et al., 2015). The bisulfite converted sperm DNA was hybridized to Illumina Infinium HumanMethylationEPIC microarrays and ran as recommended by the manufacturer (Illumina) at Infinity BiologiX.

### Data preprocessing

**FIGURE 1:**
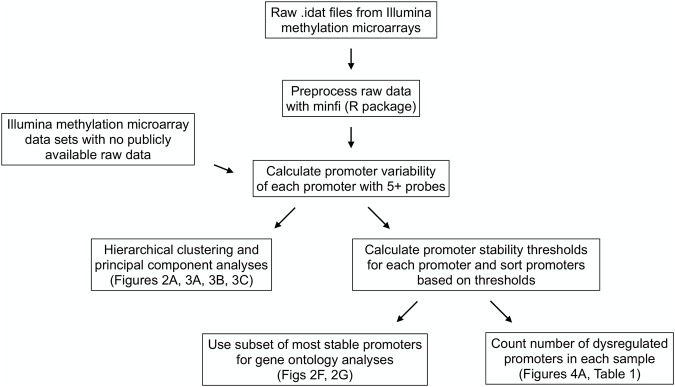
Data processing and statistical analysis workflow. Processing and analysis of Illumina’s Infinium HumanMethylation450 and HumanMethylationEPIC array data from multiple tissue types to derive promoter variability and promoter stability thresholds and analyze their relationships among tissue types and between healthy and diseased tissues.

Figure 1 contains a flow chart of data processing and statistical analysis. The raw methylation microarray data available (data from the sperm, neuron, glia, skin, CD4^+^ T cell, and CD8^+^ T cell samples) were preprocessed using the minfi R package (Aryee et al., 2014) using SWAN normalization to produce beta and m-values for each cytosine-guanine dinucleotide (CpG). Beta values are described as [methylated probe intensity/(methylated + unmethylated probe intensity + 100)] and range from 0-1 with values around 0 being unmethylated and values around 1 being methylated. M-values are described as (log (methylated probe intensity/unmethylated probe intensity) and are useful measures of methylation to prevent bias arising from heteroscedasticity seen when analyzing beta values (Du et al., 2010) (Supplemental Figure S1).

Raw data for the TCGA datasets as assembled on the UCSC Xena platform and the lung and liver datasets (GSE51077 and GSE61258, respectively) were not available, so the available beta values were used. These beta values were logit-transformed to obtain the m-values for these samples.

### Statistical analysis

We define a given gene promoter as the genomic region one kilobase upstream and one kilobase downstream from the transcription start site of a given gene. A gene promoter needed to contain five or more methylation array probes to be used in any downstream analysis. Gene methylation promoter variability (or “promoter variability”) is defined as the standard deviation of the m-values of the methylation array probes present in a defined promoter region (Supplementary Figure S2).

Hierarchical clustering was performed on all promoter variability values of samples from various tissue types using the R software package “pheatmap” (R version 4.0.3) with default parameters. In cases where more than 20 samples existed for a given tissue, 20 samples were randomly selected for inclusion in the clustering analysis to give a more uniform number of samples per tissue type. Principal component analyses were performed on all promoter variability values using the “sklearn” library in Python (Python version 3.7.3).

**FIGURE 2:**
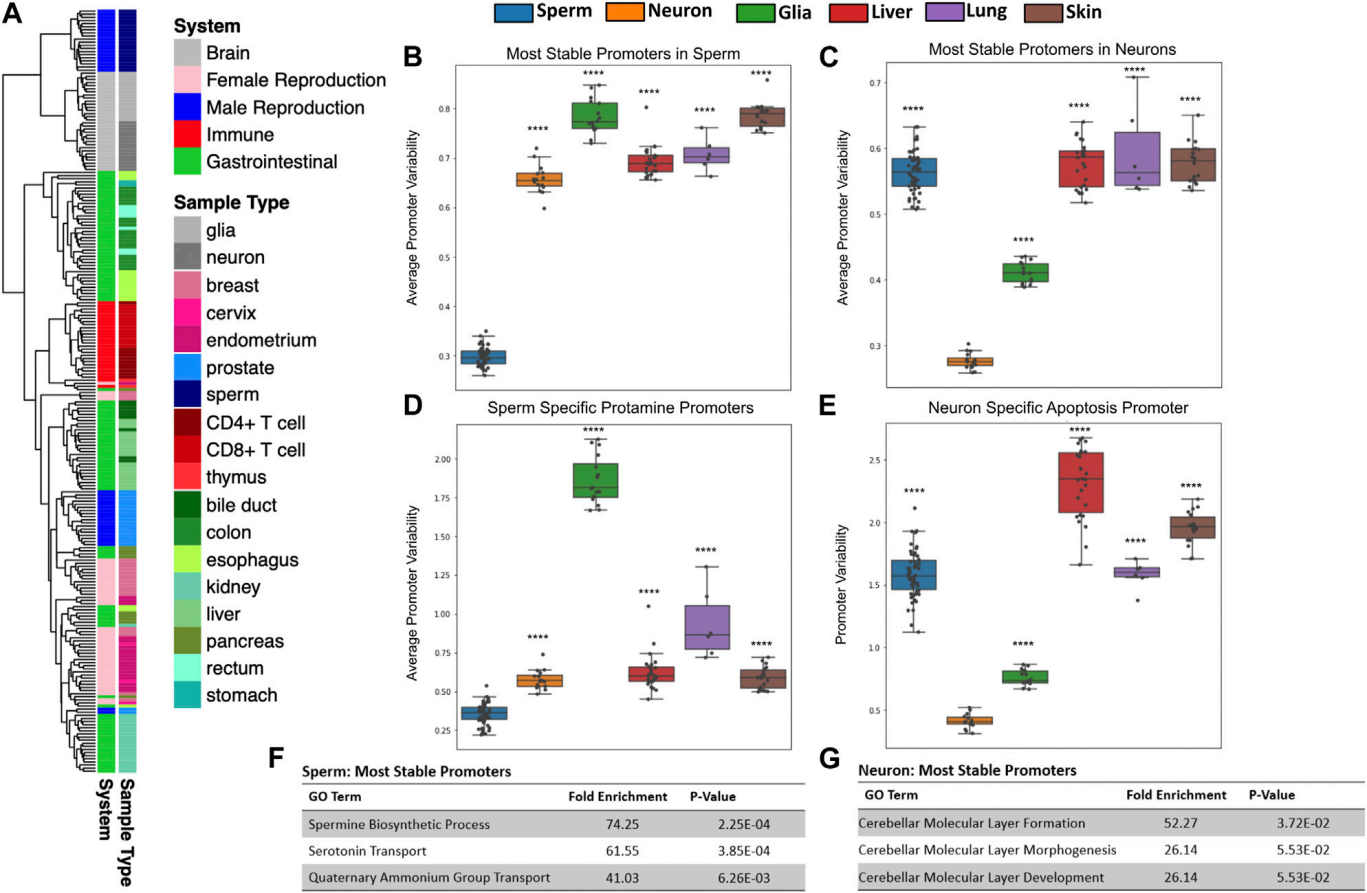
Tissues have unique patterns of gene methylation promoter variability. **(A)** Hierarchical clustering of promoter variability of 18 sample types representing five biological systems. **(B,C)** Average promoter variability of 6 distinct cell types in the most stable promoters (top first percentile) in sperm and neurons, respectively. One dot represents one sample, and boxplots are overlaid to show the distribution of average promoter variability of each tissue. All *p*-values comparing methylation variance between sperm and neuron to other tissues types were ≤5.16E-14. **(D)** Average promoter variability in the 6 cell types of three sperm-specific protamine promoters. **(E)** Promoter variability from one neuron-specific apoptosis promoter in the 6 cell types. All *p*-values for panels D and E when comparing sperm and neurons to other cell types were ≤9.99E-17. **(F,G)** Gene ontology enrichment of the most stable promoters for sperm and neurons, respectively.

We found the most epigenetically stable promoters of a given tissue type by identifying the promoters with the lowest levels of variability in healthy samples of that tissue type. We did this by first calculating a stability threshold for each promoter in a given tissue. A promoter stability threshold represents the highest level of variability we expect to see in a given promoter of a healthy sample of a given tissue (Supplementary Figure S2). Then, the promoters were rank ordered by the stability threshold values in ascending order. For the analyses comparing promoters across tissue types (Figures 2B, C, F, G), we defined the most stable promoters as the top first percentile of promoters with the lowest stability thresholds in healthy samples of the given tissue. The most stable promoters used for the analyses on sperm from men suffering from infertility were defined as the top 10th percentile of promoters with the lowest stability thresholds in fertile sperm donors.

Sperm analyses were performed by finding the most stable promoters in a cohort of fertile sperm donors and counting the number of dysregulated promoters in each sample. We defined a dysregulated promoter as a promoter that fell above the corresponding variability threshold. Samples with the lowest number of dysregulated promoters are most similar to healthy controls.

Statistical differences in the pregnancy and live birth rates of men undergoing intrauterine insemination (IUI) and *in vitro* fertilization (IVF) with the least and greatest number of dysregulated promoters were calculated with two-sided t-tests.

Tissue-specific gene ontology enrichment analyses were performed by running the PANTHER Overrepresentation Test (http://pantherdb.org/webservices/go/overrep.jsp) on the gene names of the first percentile of most stable promoters in a given tissue. Each test was run using a background gene set that consisted of all genes with promoters containing five or more methylation array probes.

The differentially methylated region (DMR) analysis between sperm of fertile sperm donors and patients with unsuccessful IUI treatments was performed using the Methylation Array Scanner application from the USeq collection of bioinformatics software (https://github.com/HuntsmanCancerInstitute/USeq). This analysis was performed on the beta values of the sperm samples from these two sample cohorts and uses a sliding window approach to identify DMRs. A cutoff was created by multiplying the average beta value of the fertile sperm donors at the specified DMR by two standard deviations.

## Results

### Tissues have unique methylation variability signatures

Using microarray DNA methylation data, we explored the differences in gene promoter methylation variability of various healthy tissues. Unsupervised clustering of all gene promoter variability values showed tissue specificity and also revealed similarities among related tissues (Figure 2A). For example, gastrointestinal tissue samples such as those from the esophagus, stomach, colon and rectum, clustered closely together. We likewise saw clustering of samples associated with the immune system (CD4^+^ T cells, CD8^+^ T cells, thymus), female reproduction (endometrium, cervix), and brain (glia, neurons).

### Lower promoter variability seen at tissue-specific biological pathways

We sought to identify how promoter variability differs among various tissue types. We identified the most stable promoters in sperm and assessed the average methylation variability values for these promoters in many samples across several tissue types as seen in Figure 2B. At promoters indicated as most stable in the male germline, sperm samples have significantly lower mean variability values than other tissue samples. Gene ontology analysis of these sperm promoters show significant enrichment for sperm-related biological processes (Figure 2F). We also looked at the mean of the promoter variability values of the known sperm-related genes protamine 1 (PRM1), protamine 2 (PRM2), and protamine 3 (PRM3) which are genes expressed exclusively in sperm and replace the majority of histones to achieve extreme nuclear compaction in this specialized cell (Balhorn, 2007). As expected, sperm samples displayed significantly less variability in these promoters than other tissues (Figure 2D). These same analyses were performed for the most stable promoters in neurons (Figures 2C, E) and a known neuron-specific gene, CASP8 (Figure 2G) with similar results. Supplementary Figure S4 contains the results of these same analyses performed for several other tissue types. It is important to note that while the most stable promoters in a given tissue are generally characterized by very low promoter variability in samples from the given tissue, these promoters all have varying degrees of absolute methylation (hypo, mid, or hyper-methylation), a feature missed by traditional differentially methylated region (DMR) analyses (Supplementary Figure S5). It is also conceivable that this method can help overcome technical biases inherent to methylation microarrays such as batch effects.

It has been shown previously that a clear relationship exists between gene promoter CpG density and absolute methylation state. More specifically, the majority of gene promoters are CpG-rich and in turn are typically unmethylated. However, the minority of gene promoters that are CpG-poor tend to have a wider range of methylation states (Bestor et al., 2014). When analyzing sperm-specific and neuron-specific promoters in sperm and neuron samples, we see this same relationship. However, we do not see a clear relationship between promoter CpG density and promoter methylation variability in these same gene promoters (Supplementary Figure S6).

### Methylation variability can differentiate between healthy and diseased tissue

**FIGURE 3:**
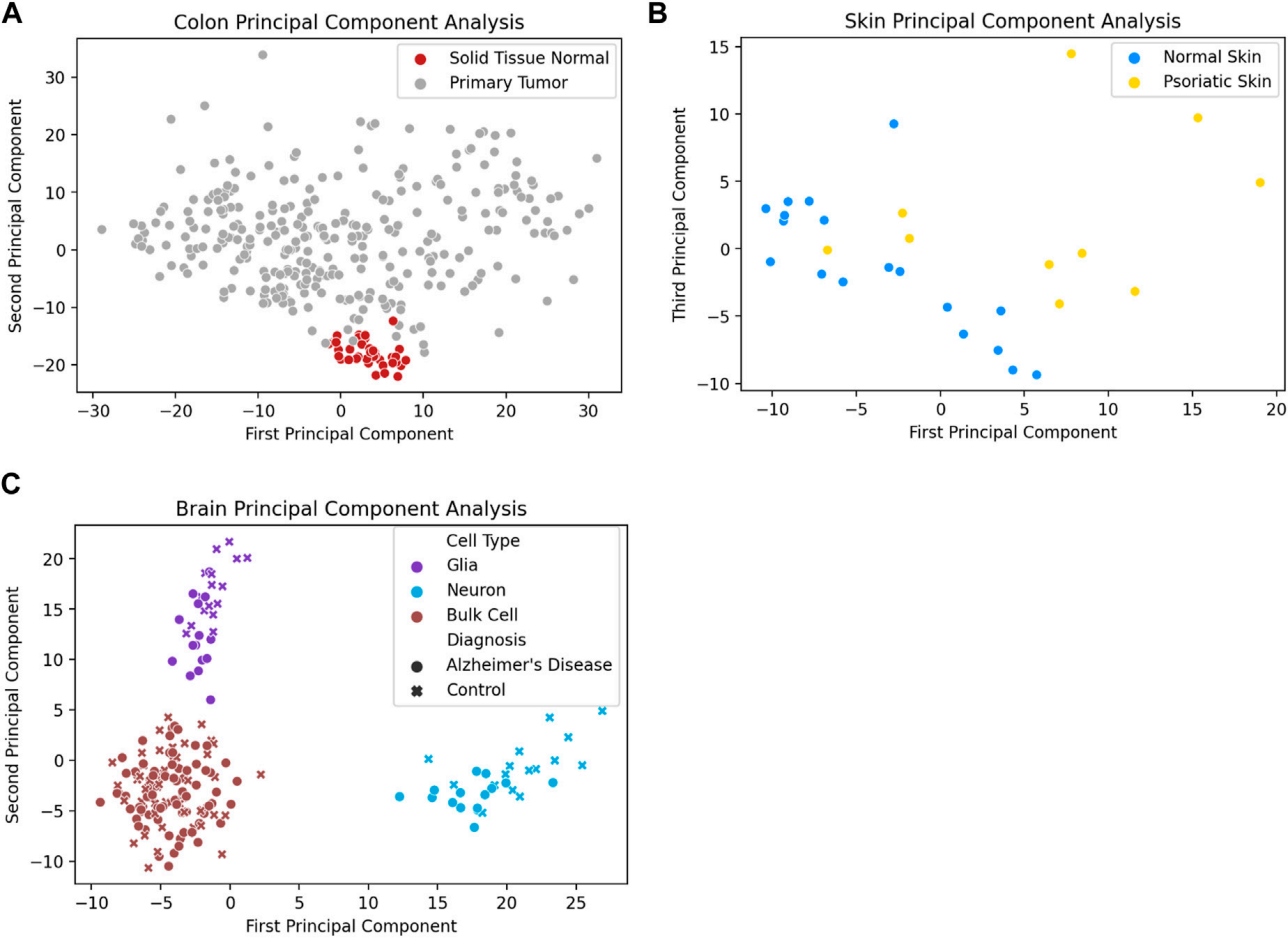
Diseased tissue samples have unique patterns of gene methylation promoter variability compared to healthy tissue samples. **(A)** Principal component analysis of promoter variability values from primary colon tumors and normal colon tissue. **(B)** Principal component analysis of promoter variability values from matched psoriatic lesion and healthy skin samples. **(C)** Principal component analysis of promoter variability among neurons, glial cells, and bulk cells from postmortem brains of individuals with Alzheimer’s disease as well as controls. The colors of the markers on the plot (purple, blue, brown) refer to the different cell types (glia cells, neuron cells, bulk cells) and the shape of the marker (circle, “x”) refer to the disease state of the sample (Alzheimer’s disease, healthy control).

In addition to distinguishing between tissue types, analysis of promoter methylation variability can enable the differentiation of diseased and healthy tissue samples of the same tissue type. One notable example is the ability to distinguish between tumor and healthy tissue based on promoter variability signatures. Figure 3A depicts the first two principal components of promoter variability values for colon primary tumor tissue and healthy colon tissue. The healthy colon tissue samples appear to be tightly clustered together, whereas the tumor samples are widely distributed throughout the plot. Figure 3B shows the difference in promoter variability between psoriatic skin lesions and adjacent healthy skin samples from the same individuals. Figure 3C shows a principal component analysis of neurons, glial cells, as well as bulk cell samples from *postmortem* brain tissue of individuals with Alzheimer’s disease and controls. The plot shows clear separation among the neurons, glial cells, and bulk cell samples indicating a difference in promoter variability among different cell types in the same tissue. There is also separation between control and Alzheimer’s disease samples in neuron and glial cell samples but such separation is not apparent among the bulk cell samples which may suggest subtle differences in promoter variability might be more apparent when samples are sorted for individual cell types.

### Methylation variability of sperm can identify a subset of men with male factor infertility

**FIGURE 4:**
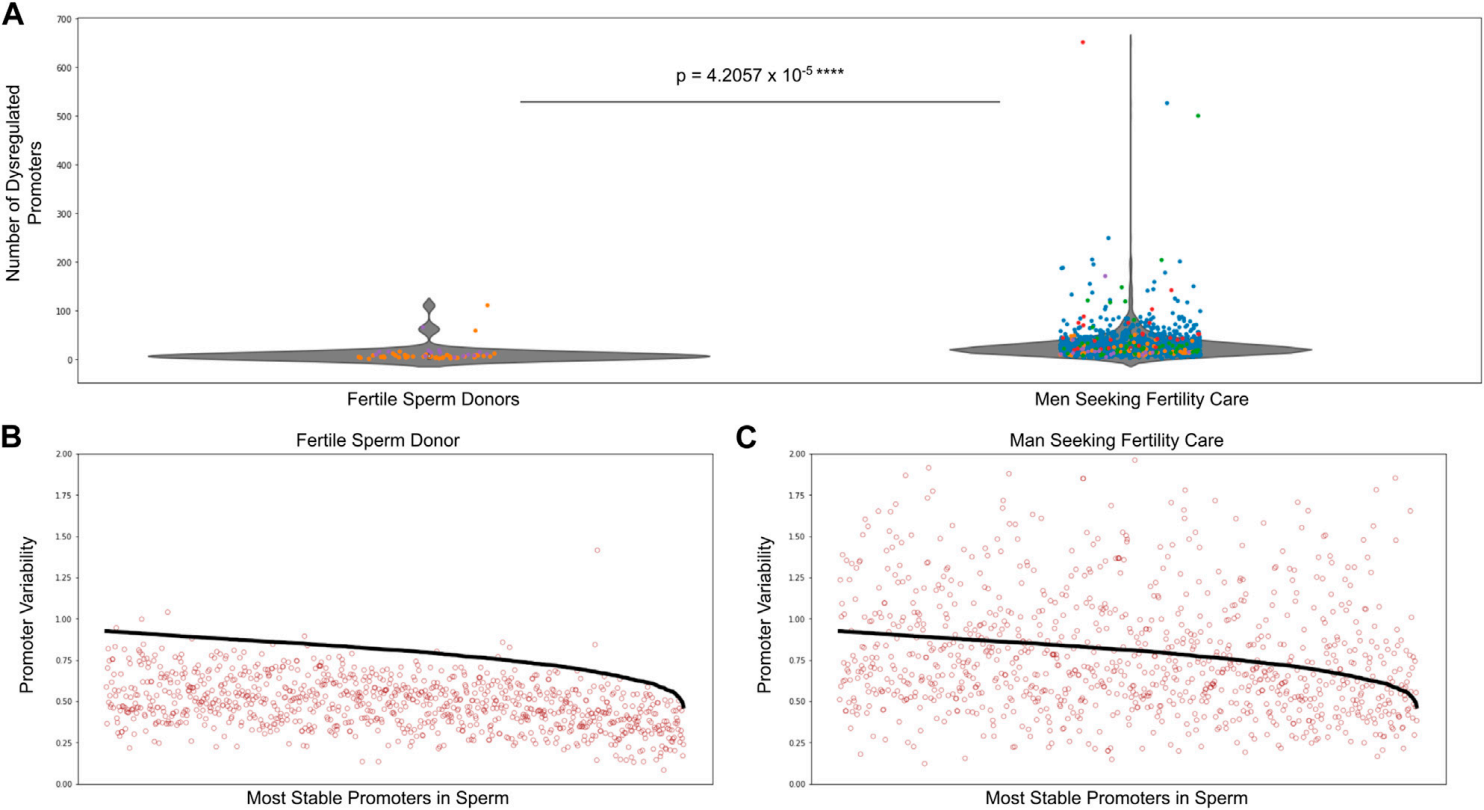
Dysregulated promoters are enriched in men seeking fertility care compared to fertile controls. **(A)** Dysregulated promoters in samples from five independent studies. The most stable sperm promoters and corresponding stability thresholds were calculated from a cohort of fertile sperm donor samples. Circle colors represent the following: blue—Jenkins et al. (Jenkins et al., 2021), green—Baylor College of Medicine, purple—fertile sperm donors, red and orange - University of Utah Andrology two independent collections. **(B)** Promoter variability at the most stable sperm promoters in a single fertile donor sperm sample (red dots). The stability threshold for these promoters is shown in black. A red dot above the black line indicates a dysregulated promoter. **(C)** Same analysis as in **(B)** but for a single patient being treated for male factor infertility.

We performed analyses on over 1,500 sperm samples from fertile sperm donors as well as men being treated for male factor infertility. Figure 4A shows there was a significantly higher number of dysregulated promoters in men being treated for male factor infertility compared to fertile sperm donors. Supplementary Figure S9 shows the number of dysregulated promoters in multiple cohorts of sperm samples, including the sperm donor cohort used to find the most stable promoters in sperm as well as the stability thresholds. To give a visual explanation of promoter methylation, Figure 4B depicts the promoter variability values (red dots) of the most stable sperm promoters and the corresponding stability thresholds for these promoters (black line) in an individual fertile sperm donor sample as well as an individual patient being treated for male factor infertility (Figure 4C). It is clear that the patient being treated for male factor infertility has many more dysregulated promoters than the fertile sperm donor. This suggests that some male factor infertility may be more related to a global shift in methylation variability at promoters important for sperm cells rather than single nucleotide changes or epimutations.

To interrogate the clinical utility of this analysis, we then looked at the relationship between dysregulated promoters in sperm compared to pregnancy and live birth rates. We analyzed the sperm-specific epigenetics of the male partner in 1,287 couples being seen by a physician for infertility care. Table 1A shows that in men from couples undergoing intrauterine insemination (IUI), those with least number of dysregulated promoters (lowest 10th percentile of patients) had significantly higher pregnancy and live birth rates than men with the greatest number of dysregulated promoters (top 10th percentile of patients). However, we saw no difference in pregnancy and live birth rates of couples undergoing *in vitro* fertilization (IVF) where the male partner had either the least or greatest number of dysregulated promoters, suggesting IVF should be the preferred fertility treatment option for men with high levels of methylation dysregulation.

**TABLE 1:** Assisted reproductive technology outcomes and gene promoter variability in sperm. A) Pregnancy and live birth rates from couples undergoing intrauterine insemination (IUI) cycles (cumulative average of 2-3 cycles across the patient population) where the male partner had either among the least or the greatest number of dysregulated promoters in sperm. B) Pregnancy and live birth rates from couples undergoing *in vitro* fertilization (IVF) cycles (cumulative average of 2-3 cycles across the patient population) where the male partner had either among the least or the greatest number of dysregulated promoters in sperm.

A		
Patient cohort	Pregnancy rate from IUI	Live birth rate from IUI
Male partners (*N* = 54) with among least number of dysregulated promoters (bottom 10th percentile)	48.1%	40.7%
Male partners (*N* = 50) with among greatest number of dysregulated promoters (top 10th percentile)	28.0%	18.0%
	*p* = 0.035	*p* = 0.011

B		
Patient cohort	Pregnancy rate from IVF	Live birth rate from IVF
Male partners (*N* = 36) with among least number of dysregulated promoters (bottom 10th percentile)	75.0%	61.1%
Male partners (*N* = 24) with among greatest number of dysregulated promoters (top 10th percentile)	75.0%	62.5%
	*p* = 1.00	*p* = 0.915

To compare this new method of methylation analysis to current methods, we performed a differentially methylated region analysis between sperm from fertile men and men from couples failing to conceive with IUI. The largest DMR found was in the gene SNORD115 (chr15: 25425615-25494878, GRCh37) with the cohort of fertile men having lower mean methylation levels of CpGs in this DMR than men from couples failing IUI (Supplementary Figure S10). We then created a cutoff two standard deviations above the average methylation value of the fertile cohort at this DMR. Men with a mean methylation value above this cutoff at this DMR would be predicted to be less fertile than men with a mean methylation value below the cutoff. Then, we examined the methylation of this DMR in the separate cohort of 1,287 couples seeking infertility care. In this large cohort, we saw no difference in the pregnancy and live birth rates between couples receiving IUI regardless of the methylation state of this DMR in SNORD115 in the male partner’s sperm (Table 2).

**TABLE 2:** Intrauterine insemination (IUI) outcomes and methylation state of IUI sperm DMR. Pregnancy and live birth rates from couples undergoing intrauterine insemination (IUI) cycles (cumulative average of 2-3 cycles across the patient population) where the male partner had a methylation state below or above the methylation cutoff of the SNORD115 DMR in sperm.

Patient cohort	Pregnancy rate from IUI	Live birth rate from IUI
Methylation of male partner’s (*N* = 467) sperm sample was below DMR cutoff	45.0%	32.5%
Methylation of male partner’s (*N* = 61) sperm sample was above DMR cutoff	47.5%	36.1%
	*p* = 0.705	*p* = 0.583

## Discussion

Here, we introduce a novel method to assess complex disease. This method analyzes gene promoter DNA methylation variability to identify highly regulated genes in multiple tissue types and how these can be impacted in various disease states. Hierarchical clustering of gene promoter variability from many tissues demonstrates how unique these patterns are in different tissues and how these patterns remain largely consistent in related, but distinct tissues. For example, numerous tissues from the gastrointestinal tract cluster together as do tissues important to the function of the immune system.

We also found that the most stable promoters in a given tissue have significantly lower methylation variability than the same promoters in other tissues highlighting the importance of unique genes and gene networks to any given tissue’s function. Importantly, when assessing DNA methylation variability within the same tissue type, we were also able to visualize differences between healthy and diseased tissues such as in cancer, psoriasis, and Alzheimer’s disease.

To highlight the potential clinical impact of the assessment of promoter level DNA methylation variability, we examined the pattern’s utility in an assessment of male factor infertility and found that the sperm of men being seen by a physician for infertility had much higher levels of dysregulated promoters. In addition, couples undergoing IUI treatments where the man had a greater number of dysregulated promoters had significantly lower pregnancy and live birth rates compared to couples undergoing IUI treatments where the male partner had a low number of dysregulated promoters. However, this stark difference in pregnancy and live birth rates was not seen between couples receiving IVF. This finding has great clinical significance because it suggests that if a man is struggling with infertility and has a high level of promoter dysregulation, he has much better odds at having a child with his partner after undergoing IVF than if they simply went through multiple rounds of IUI. We do note that men from couples who were unable to conceive a child through IUI had slightly lower total motile sperm counts than men who conceived a child through IUI, however the difference was small and not statistically significant (Supplemental Tables 5A, 5C).

Importantly, our additional analysis of a more conventional approach to identify a clinically actionable diagnostic based on targeted differential methylation failed to result in significant findings when applied to a large independent cohort. In contrast, our approach to identify methylation variability across genes important in sperm were indicative of clinically meaningful phenotypes. We believe that assessing DNA methylation in this way will offer improved predictive power for many disease states because it is focused on more widespread alterations to gene regulation as opposed to single site alterations.

Of additional value in our assessment of this approach and its translational capacity is the ability to perform these analyses at an individual level. Specifically, because we are assessing intra-promoter variability within a single individual, we are able to reliably assess variability in a single individual with limited concerns of batch effects which would require normalization. While this has yet to be fully vetted in the current work, it does appear that these analyses, if proven to have high predictive power, could be translated to a diagnostic tool.

While we did perform one of the largest analyses to date in terms of tissue types and sample numbers, many questions still remain to be answered. Among the most critical is the utility of this analysis in many different tissues and disease states. Because we were only able to perform a deep analysis in sperm due to the large numbers of samples, similar work needs to be done in other tissues to determine if this analysis can provide clinically actionable information. Even in our sperm study, this work needs to be replicated in independent cohorts to determine its efficacy. One area of concern for this type of analysis is the fact that not all tissues are able to be purified as easily as sperm. In fact, we found that when looking at bulk tissues in the brain, we lost any ability to discriminate between healthy and diseased tissue. Thus, future work needs to focus on as pure of tissue as possible to yield meaningful results. We also believe future work with single cell methylation detection technologies will be useful to identify subpopulations of cells that give rise to a disease phenotype. In addition, these types of future studies could help identify and study possible mechanisms of disease.

These findings provide a novel means to define which genes each cell and tissue type tightly regulate to ensure their unique phenotype and function. Because these signals have potential utility in both the basic understanding of tissue-specific epigenetic patterns and in the clinical assessment of diseased tissues, as well as the prediction of outcomes, this work provides important foundational findings upon which tissue and disease-specific assessments can be constructed in the future. While much work remains to determine clinical actionability for various applications, the results here are encouraging and may offer another tool with which we can assess the health of tissues and, importantly, predict the outcomes from various clinical interventions.

## Data Availability

The source of the data presented in the study are included in the article. Further inquiries can be directed to the corresponding author.
