# Supplementary material for: Tissue-specific DNA methylation variability and its potential clinical value
Source: Front Genet. 2023 Jul 19;14:1125967. doi: 10.3389/fgene.2023.1125967 (PMC10394514; doi:10.3389/fgene.2023.1125967)
Supplement: Supplementary file 1 [file DataSheet1.PDF]

Supplemental Figure 1

Heteroscedasticity of beta values in a sperm donor sample. A) Distribution of mean promoter methylation of the 100 most stable promoters in sperm which were found by calculating the variability value of the beta values of all probes in a promoter region. **B)** Distribution of mean promoter methylation of the 100 most stable promoters in sperm which were found by calculating the variability value of the m-values of all probes in a promoter region.

A

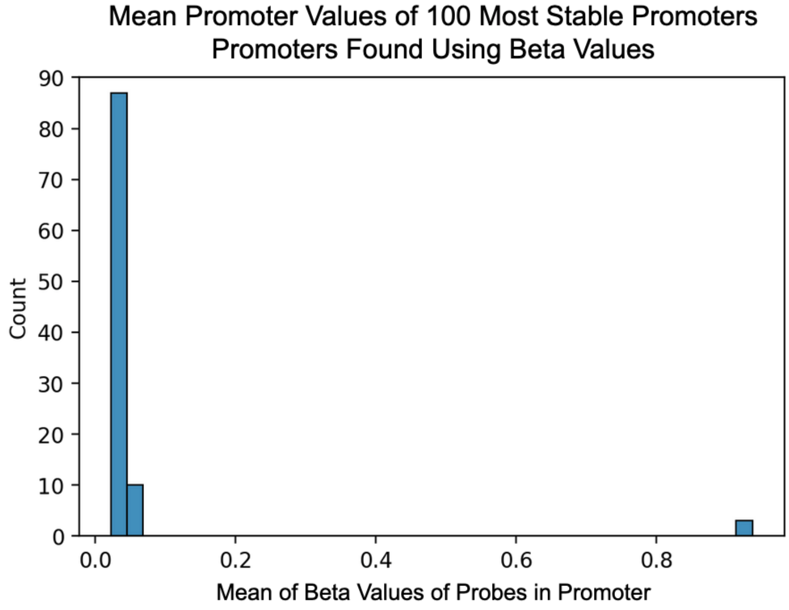

B

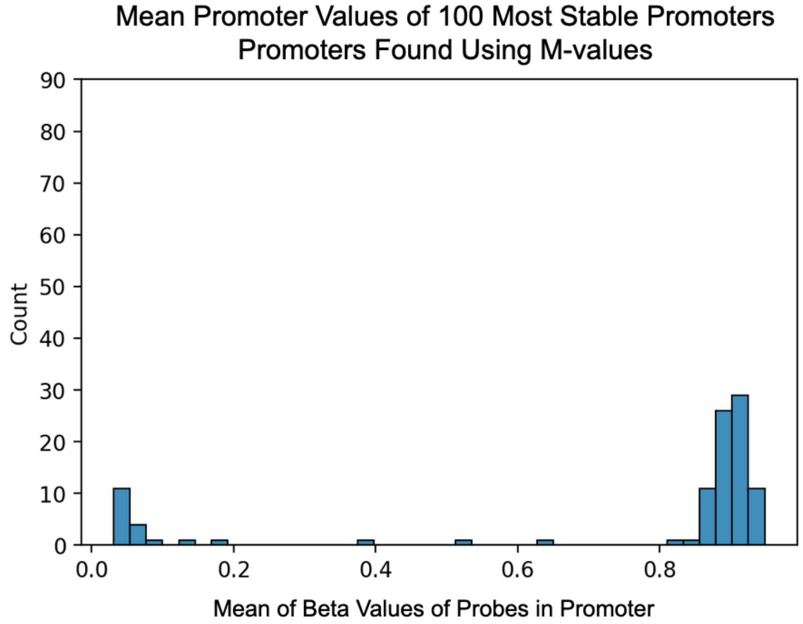

Supplemental Figure 2

Variability equations. A) Equation for calculating the variability value (or standard deviation) of a given promoter in a sample; σ = gene promoter variability value, x_1 = m-value of a given methylation array probe in a given promoter, μ = mean of probe m-values in given promoter. **B)** Equation to calculate the promoter variability threshold for a given tissue. Θ = promoter variability threshold for a given tissue, σ_1 = promoter variability value of a sample in a given cohort at a given promoter, μ = mean of the promoter variability values at a given promoter in a given cohort.

A

$$\sigma = \sqrt{\frac{\sum |x_1 - \mu|^2}{N}}$$

B

$$\theta = \frac{\sum \sigma_1}{N} + 3\sqrt{\frac{\sum |\sigma_1 - \mu|^2}{N}}$$

Supplemental Figure 3

Hierarchical clustering of different control tissues using beta values. This plot was created by performing hierarchical clustering on all promoter mean values of the beta values of probes in a given promoter.

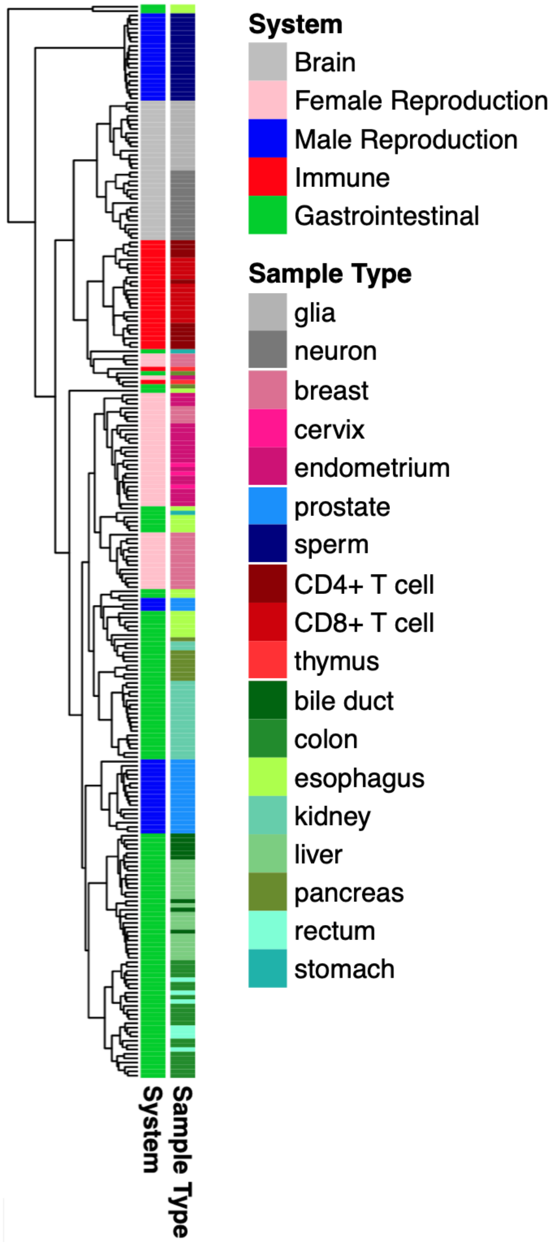

Supplemental Figure 4

Average promoter variability at tissue-specific promoters. A) Average promoter variability values of numerous samples across several tissues at the most stable promoters in control lung tissue. **B)** Average promoter variability values of numerous samples across several tissues at the most stable promoters in control skin tissue. **C)** Average promoter variability values of numerous samples across several tissues at the most stable promoters in control liver tissue.

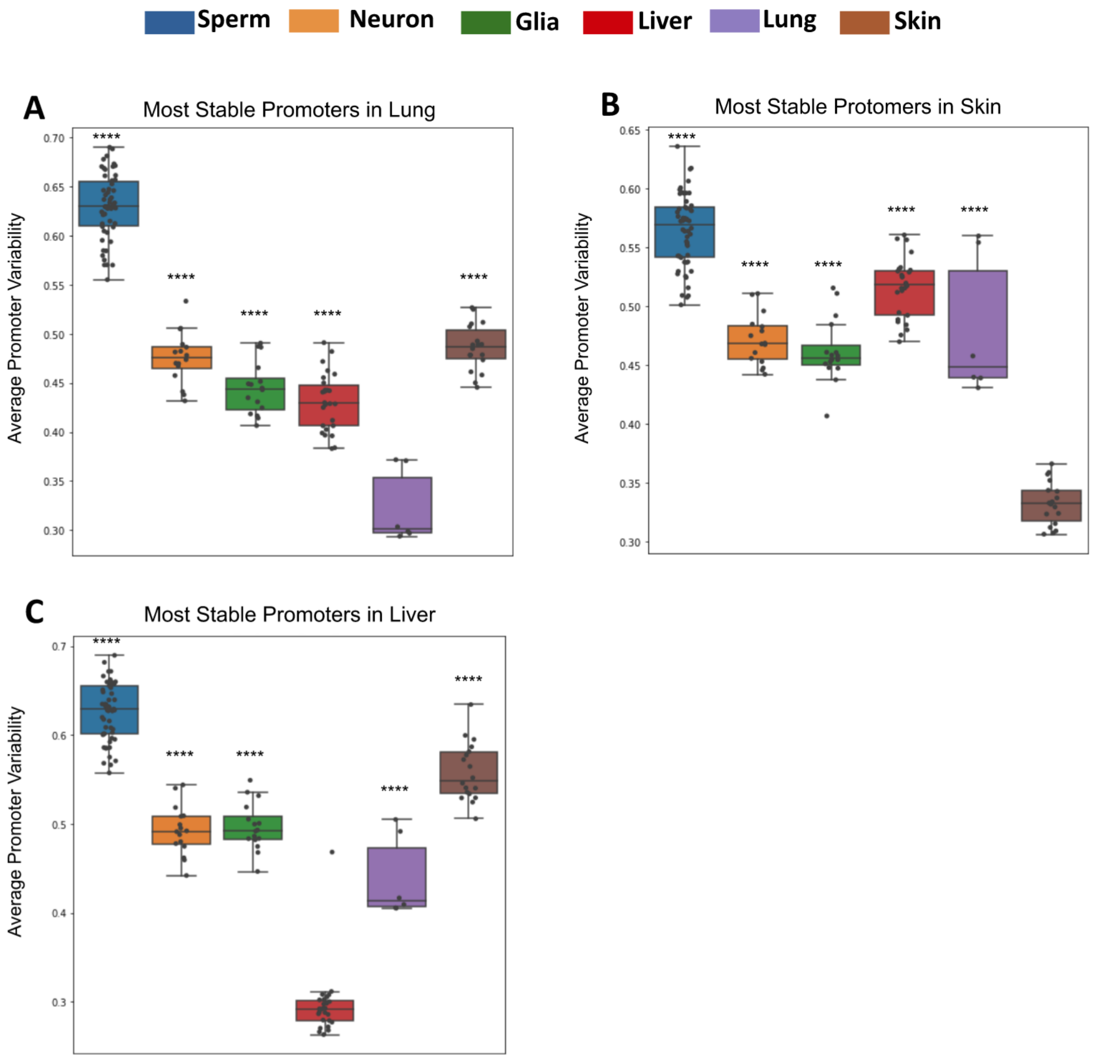

Supplemental Figure 5

Example of promoters with low methylation variability but varying levels of methylation.

A) Box plot of methylation values of 31 sperm donor samples at 3 gene promoters with low methylation variability. **B)** Dot plot of methylation values of 1 sperm donor sample at 3 gene promoters with low methylation variability.

A

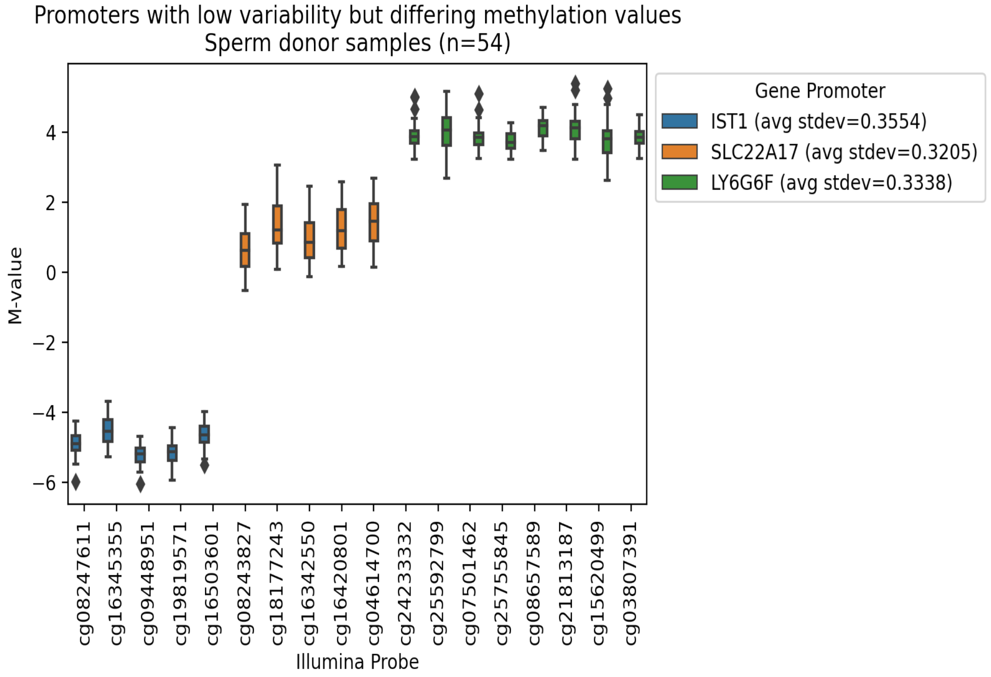

B

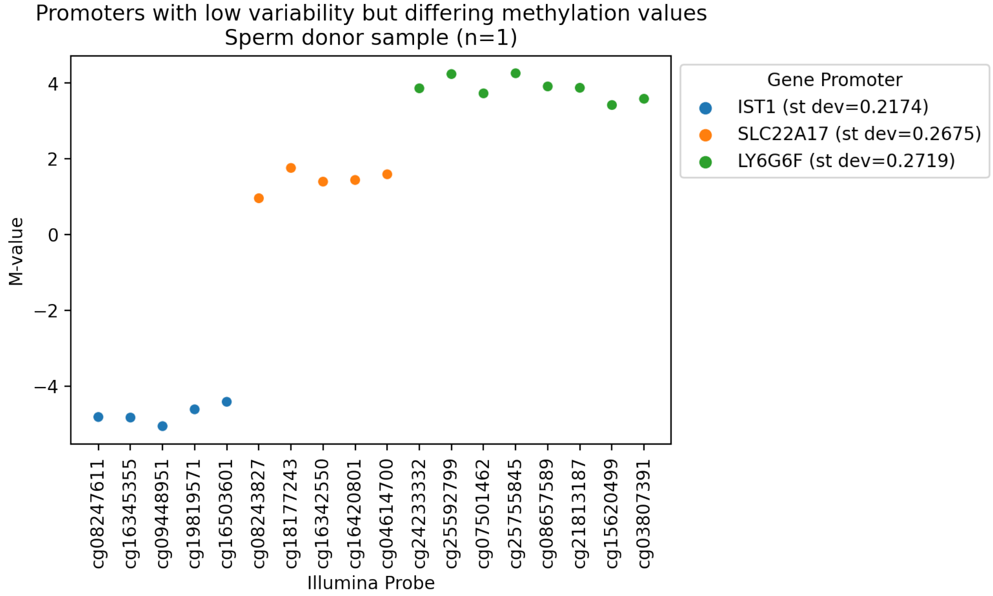

Supplemental Figure 6

Relationship of promoter CpG density to promoter methylation values (hypo, hypermethylation) and promoter methylation variability in healthy tissues. In these plots, the promoter methylation mean values refer to the average of the mean beta values of methylation array probes in the most stable promoters in the given tissue types (top 10th percentile of promoters with lowest variability). The promoter methylation variability values refer to the average of the variability values (standard deviation) of methylation array probes in the most stable promoters in the given tissue types (top 10th percentile of promoters with lowest variability).

A) Relationship between promoter CpG density and promoter methylation values of sperm-specific promoters in sperm samples. **B)** Relationship between promoter CpG density and promoter methylation values of neuron-specific promoters in neuron samples. **C)** Relationship between promoter CpG density and promoter methylation variability values of sperm-specific promoters in sperm samples. **D)** Relationship between promoter CpG density and promoter methylation variability values of neuron-specific promoters in neuron samples.

A) R^2=0.5495; p-value=3.0186x10^{-181}

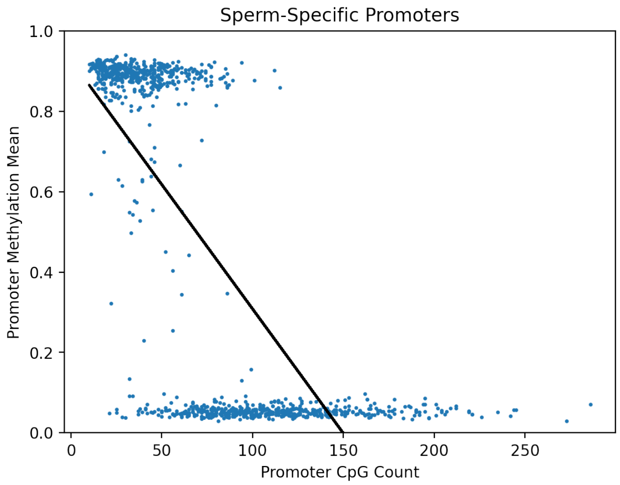

B) R^2=0.4664; p-value=3.3053x10^{-143}

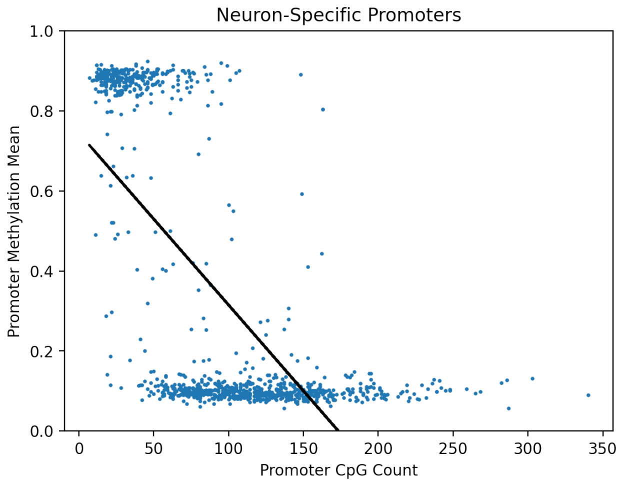

C) R^2= 0.1093; p-value=7.4370x10^{-28}

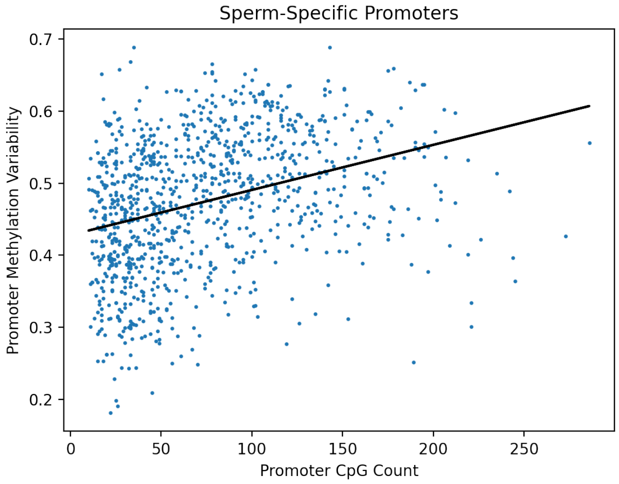

D) R^2= 0.0977; p-value=6.6063x10^{-25}

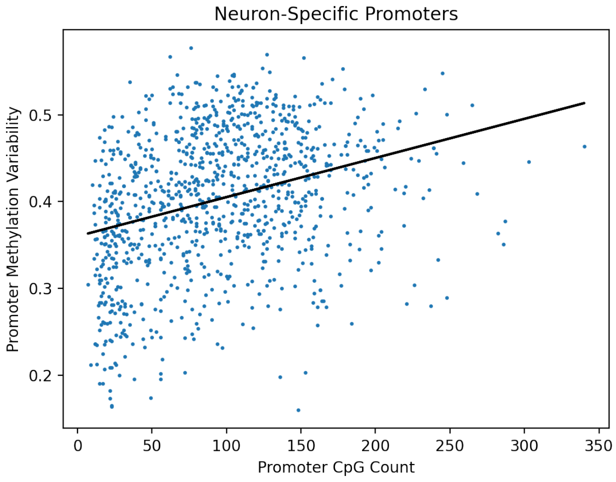

Supplemental Figure 7

Principal component analysis of diseased and control samples. This plot shows the principal component analysis of liver samples from healthy individuals and those with nonalcoholic fatty liver disease (NAFLD) and nonalcoholic steatohepatitis (NASH).

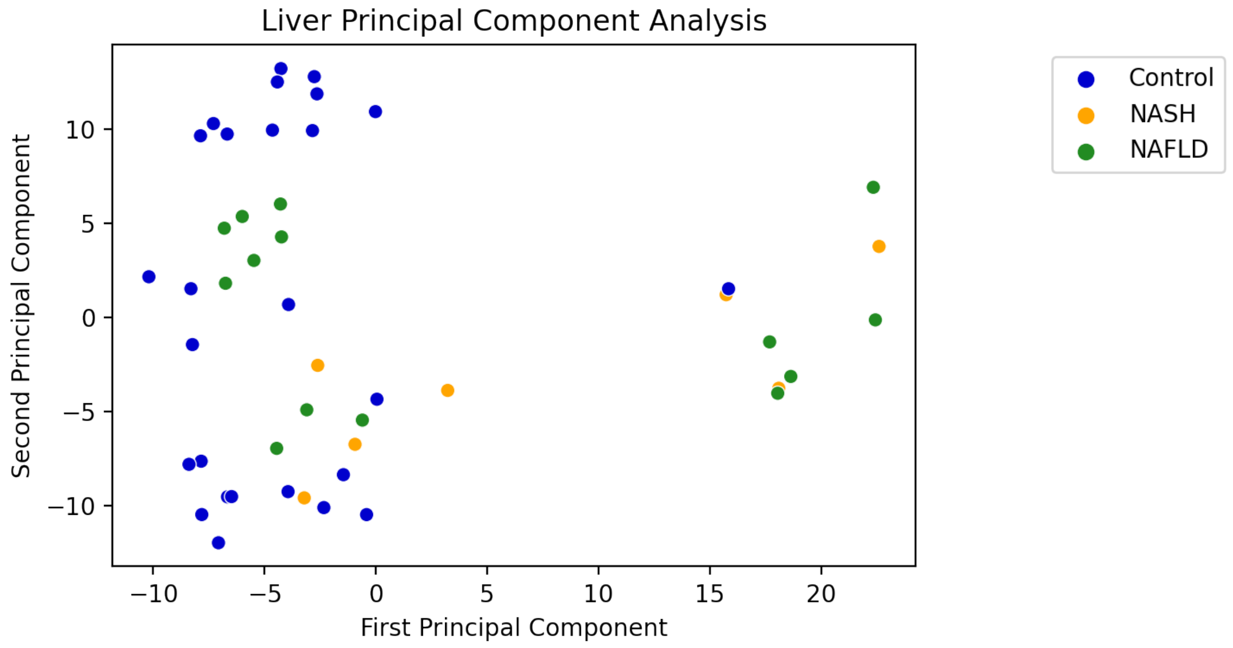

Supplemental Figure 8

Hierarchical clustering of diseased and control tissue samples. This plot shows the hierarchical clustering of diseased and control tissue samples. Clustering is based on the variability values of all promoters. **A)** Hierarchical clustering of colon primary tumor samples and normal colon tissue samples. **B)** Hierarchical clustering of paired psoriatic skin lesion samples and normal skin samples. **C)** Hierarchical clustering of control, nonalcoholic fatty liver disease (NAFLD), and nonalcoholic steatohepatitis (NASH) liver samples.

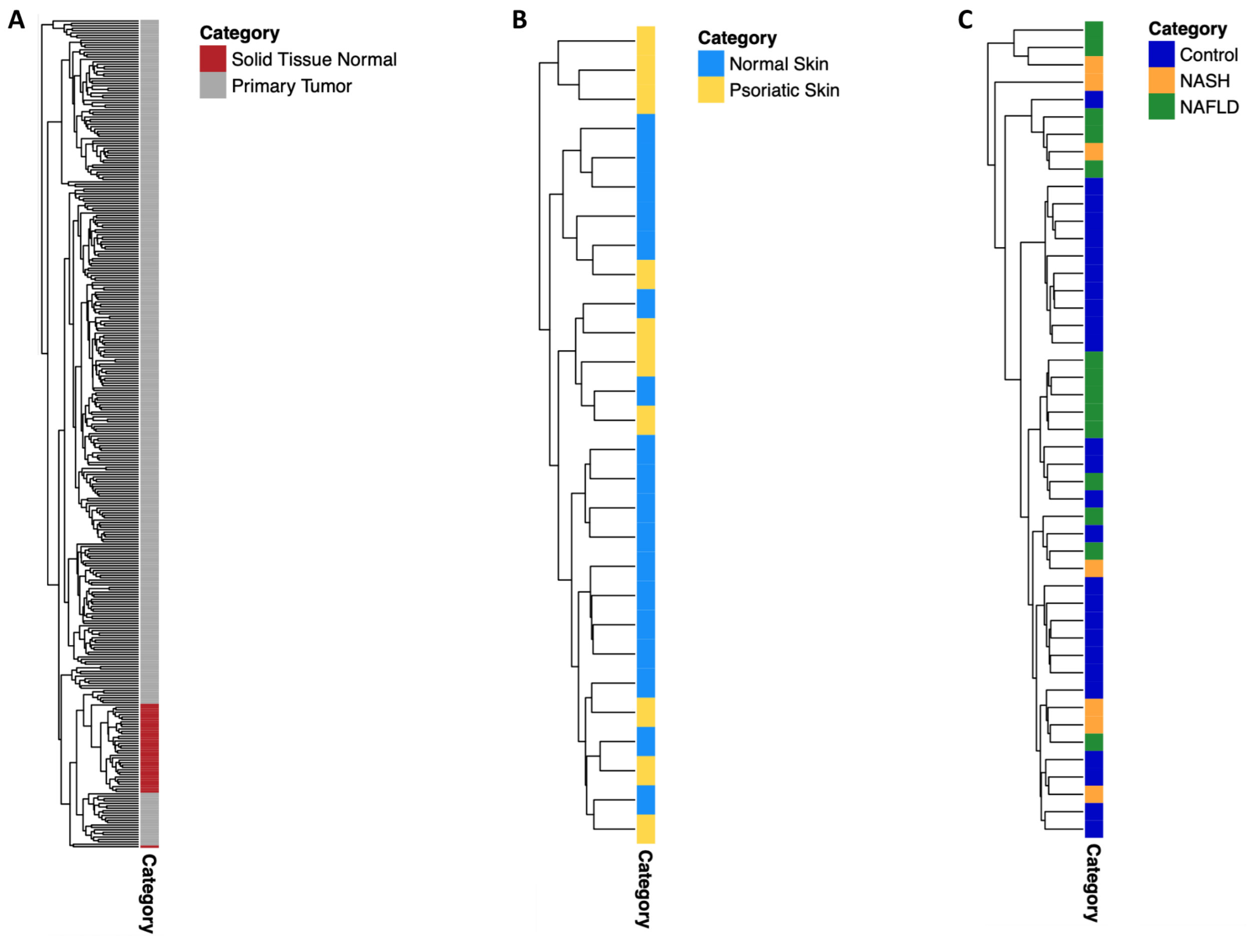

Supplemental Figure 9

Dysregulated promoter analysis of sperm samples from multiple cohorts. These plots look at the number of dysregulated promoters from the most stable promoters in sperm. The "Sperm donor cohort (training)" is the cohort used to find the most stable promoters in sperm and set the promoter variability thresholds. Panel A shows the number of dysregulated promoters in various sperm sample cohorts on a linear scale and Panel B shows the same plot but on a logarithmic scale.

A

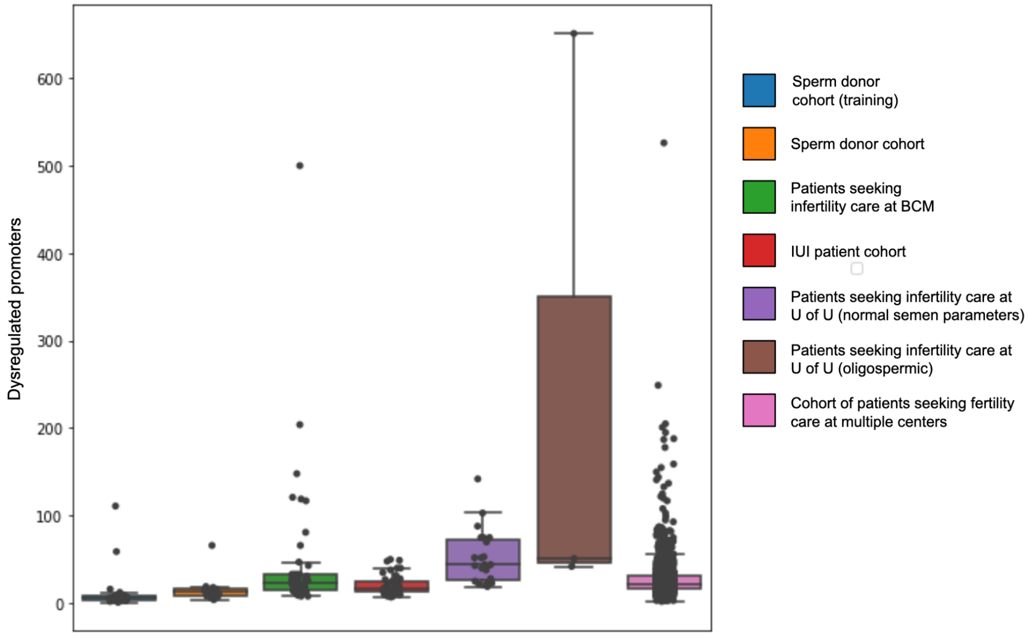

B

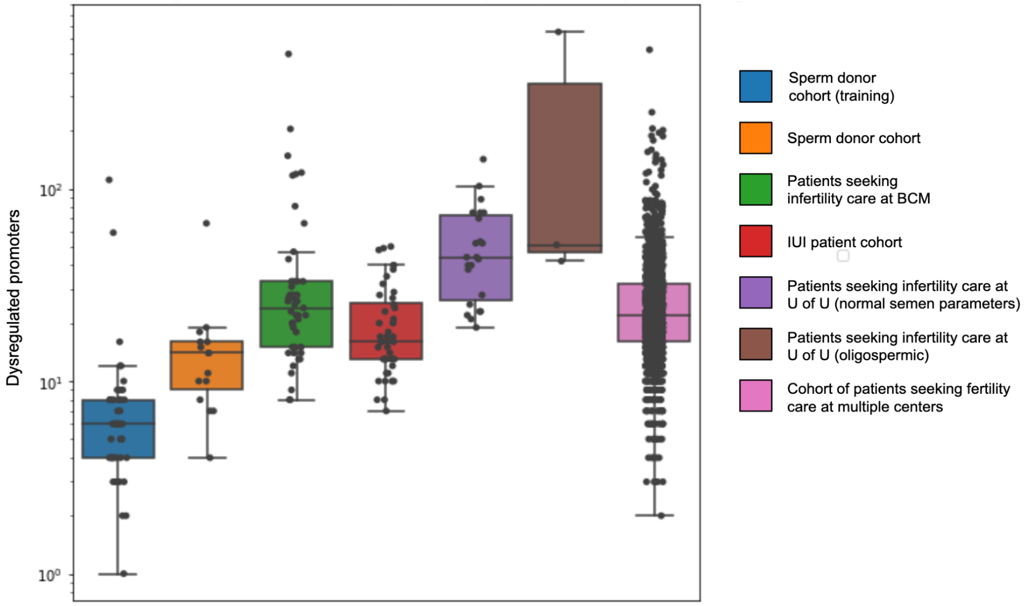

Supplemental Figure 10
Methylation levels of SNORD115

This plot shows the methylation levels (mean of beta values) of the differentially methylated region in sperm of SNORD115 (chr15:25425615-25494878, GRCh37) in a cohort of fertile sperm donors and men from couples failing to conceive with IUI. The dashed red line represents two standard deviations above the mean of the average methylation value of this DMR in the fertile donor cohort.

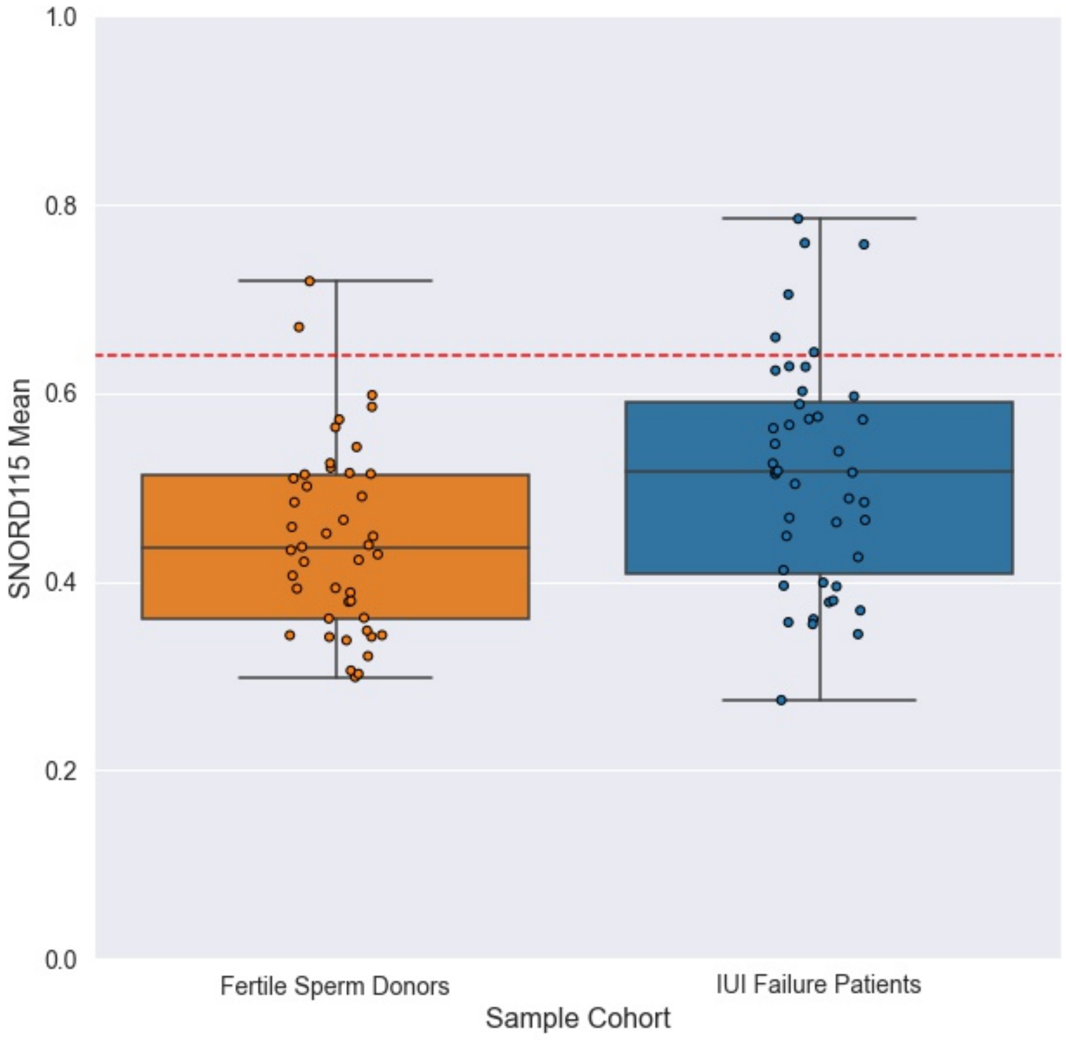

Supplemental Figure 11
Methylation levels of SNORD115 and pregnancy from IUI
This plot shows the methylation levels (mean of beta values) of the differentially methylated region in sperm of SNORD115 (chr15:25425615-25494878, GRCh37) in males from couples receiving IUI (N=528) and the result of the IUI procedures in producing a pregnancy. These men are separate from the patients shown in Supplemental Figure 10. The dashed red line represents the DMR cutoff established in Supplemental Figure 10.

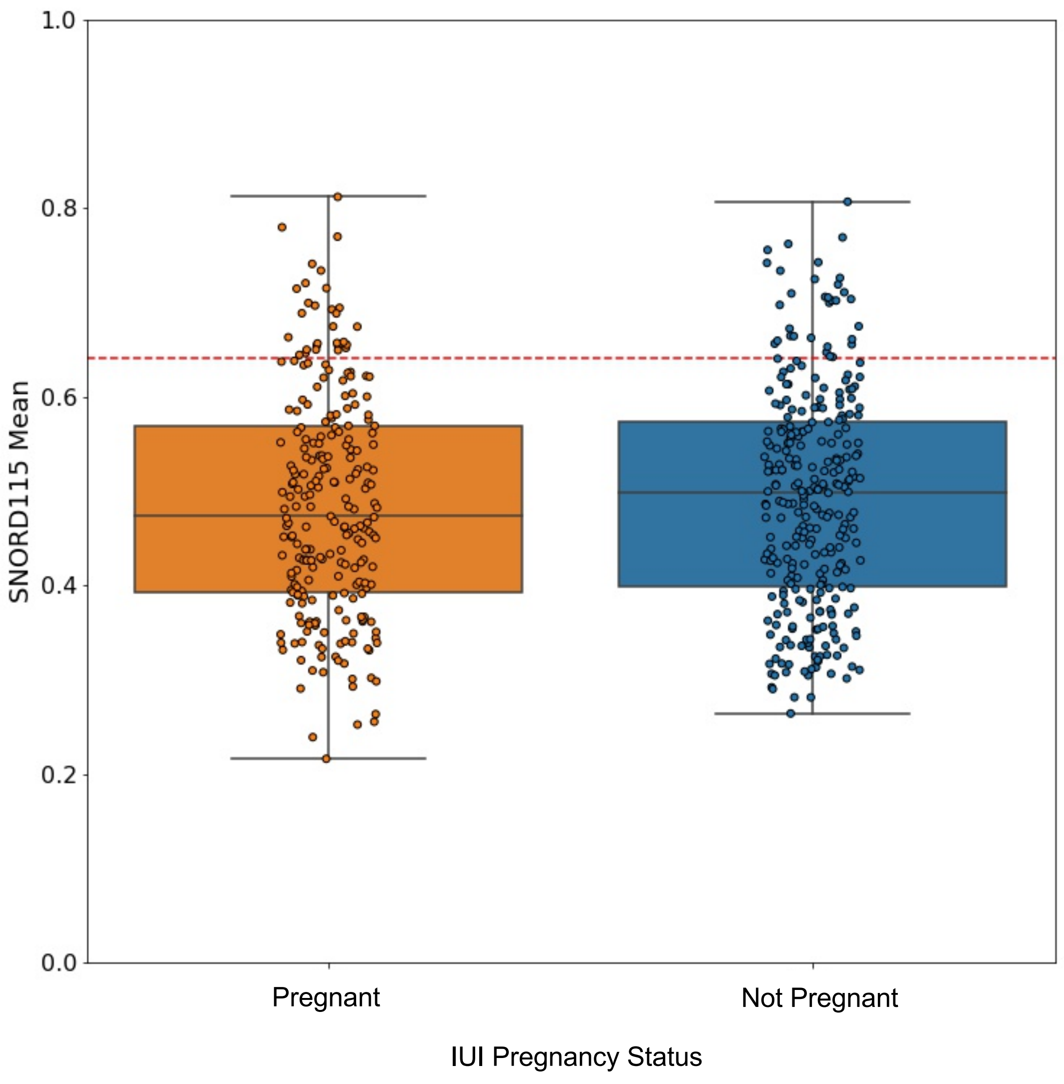

Supplemental Figure 12
Methylation levels of SNORD115 and live birth from IUI
This plot shows the methylation levels (mean of beta values) of the differentially methylated region in sperm of SNORD115 (chr15:25425615-25494878, GRCh37) in males from couples receiving IUI (N=528) and the result of the IUI procedures in producing a live birth. These men are separate from the patients shown in Supplemental Figure 10. The dashed red line represents the DMR cutoff established in Supplemental Figure 10.

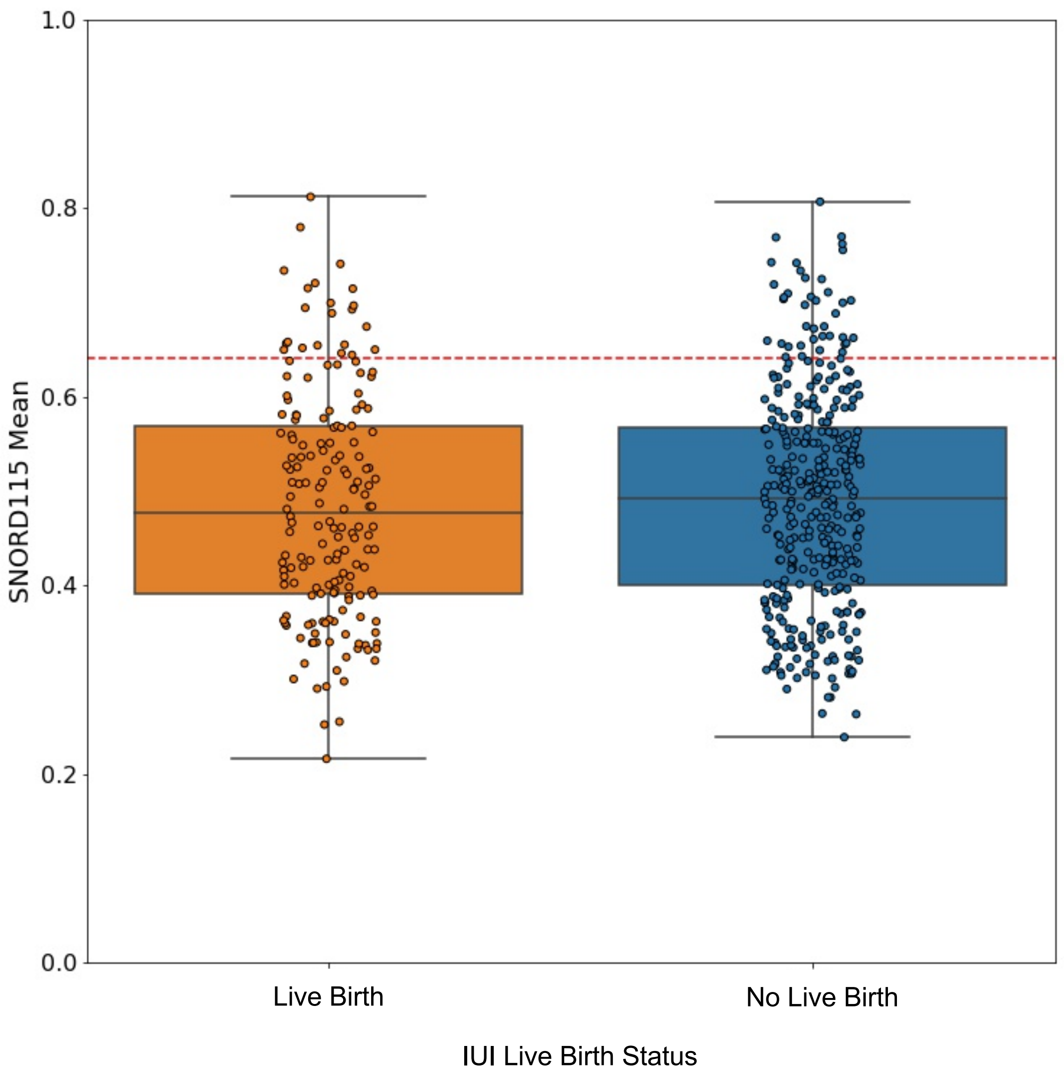

Supplemental Table 1
Promoter CpG density of genes analyzed in Figures 2D and 2E

Promoter	Promoter CpG Count	Gene Group
PRM1	37	Sperm specific protamine gene
PRM2	55	Sperm specific protamine gene
PRM3	52	Sperm specific protamine gene
CASP8	29	Neuron specific apoptosis gene

Supplemental Table 2. IUI success rates from male partners with normal sperm concentration values (≥ 15 million sperm / mL).

Patient Cohort	Pregnancy Rate from IUI	Live Birth Rate from IUI
**Male partners with among least number of dysregulated promoters (bottom 10th percentile)**	47.1%	39.2%
**Male partners with among greatest number of dysregulated promoters (top 10th percentile)**	30.2%	18.6%
**P-Value**	0.098	0.030

Supplemental Table 3. Statistics of men seeking fertility care (N=1287) with the least and greatest number of dysregulated promoters.

Patient Cohort	Mean Age (Years)	Median Age (Years)	Mean Sperm Conc (M/mL)	Median Sperm Conc (M/mL)	Mean BMI	Median BMI
**Male partners with among least number of dysregulated promoters (bottom 10th percentile)**	33.3	33.5	87.4	74.5	29.7	28.7
**Male partners with among greatest number of dysregulated promoters (top 10th percentile)**	33.5	32.0	86.3	58.5	29.2	28.0
**P-Value**	0.705		0.909		0.507	

Supplemental Table 4. Ranking by sperm concentration.

Concentration Level	Pregnancy Rate from IUI	Live Birth Rate from IUI
**Male partners with among the highest sperm concentration (top 10th percentile)**	32.1%	23.2%
**Male partners with among the lowest sperm concentration (bottom 10th percentile)**	28.6%	23.8%
**P-Value**	0.285	0.587

Supplemental Table 5a. IUI pregnancy and sperm metrics.

	Concentration (M/mL) Mean	Total Motile Count Mean	Morphology Mean
**IUI (pregnancy)**	106.11	249.49	6.73
**IUI (no pregnancy)**	96.37	215.99	5.97
**P-value**	0.234	0.140	0.060

Supplemental Table 5b. IVF pregnancy and sperm metrics.

	Concentration (M/mL) Mean	Total Motile Count Mean	Morphology Mean
**IVF (pregnancy)**	85.26	185.43	5.87
**IVF (no pregnancy)**	89.26	185.16	6.94
**P-value**	0.770	0.994	0.200

Supplemental Table 5c. IUI live birth and sperm metrics.

	Concentration (M/mL) Mean	Total Motile Count Mean	Morphology Mean
**IUI (live birth)**	106.16	256.63	6.41
**IUI (no live birth)**	98.11	218.53	6.27
**P-value**	0.359	0.112	0.743

Supplemental Table 5d. IVF live birth and sperm metrics.

	Concentration (M/mL) Mean	Total Motile Count Mean	Morphology Mean
**IVF (live birth)**	82.34	185.13	6.15
**IVF (no live birth)**	91.95	185.75	5.98
**P-value**	0.395	0.982	0.805

P-values in Supplemental Tables 5a-5d were calculated using two-sided, independent t-tests

Supplemental Table 6a. IUI pregnancy and health measurements.

	Male Age Mean	Male BMI Mean	Male Systolic Blood Pressure Mean	Male Diastolic Blood Pressure Mean	Female Age Mean	Female BMI Mean	Female Systolic Blood Pressure Mean	Female Diastolic Blood Pressure Mean
**IUI (pregnancy)**	32.21	29.02	125.77	77.98	30.63	27.61	114.68	72.72
**IUI (no pregnancy)**	32.91	29.87	126.54	78.09	31.19	28.39	114.56	74.07
**P-value**	0.125	0.127	0.497	0.910	0.189	0.269	0.921	0.143

Supplemental Table 6b. IVF pregnancy and health measurements.

	Male Age Mean	Male BMI Mean	Male Systolic Blood Pressure Mean	Male Diastolic Blood Pressure Mean	Female Age Mean	Female BMI Mean	Female Systolic Blood Pressure Mean	Female Diastolic Blood Pressure Mean
**IVF (pregnancy)**	34.37	28.51	126.40	77.94	32.11	25.74	112.96	70.53
**IVF (no pregnancy)**	36.45	28.70	127.17	78.71	35.49	27.50	113.47	70.61
**P-value**	0.029	0.828	0.683	0.623	2.70×10^{-6}	0.094	0.810	0.956

Supplemental Table 6c. IUI live birth and health measurements.

	Male Age Mean	Male BMI Mean	Male Systolic Blood Pressure Mean	Male Diastolic Blood Pressure Mean	Female Age Mean	Female BMI Mean	Female Systolic Blood Pressure Mean	Female Diastolic Blood Pressure Mean
**IUI (live birth)**	31.80	28.67	125.65	77.82	30.44	27.14	114.67	72.03
**IUI (no live birth)**	32.98	29.89	126.45	78.15	31.18	28.48	114.59	74.15
**P-value**	0.015	0.039	0.505	0.736	0.097	0.071	0.953	0.030

Supplemental Table 6d. IVF live birth and health measurements.

	Male Age Mean	Male BMI Mean	Male Systolic Blood Pressure Mean	Male Diastolic Blood Pressure Mean	Female Age Mean	Female BMI Mean	Female Systolic Blood Pressure Mean	Female Diastolic Blood Pressure Mean
**IVF (live birth)**	34.52	28.62	126.66	77.83	32.36	25.70	113.15	70.16
**IVF (no live birth)**	35.24	28.43	126.40	78.52	33.52	26.75	112.93	71.14
**P-value**	0.369	0.796	0.867	0.595	0.058	0.232	0.905	0.451

P-values in Supplemental Tables 6a-6d were calculated using two-sided, independent t-tests

Supplemental Table 7a. IUI pregnancy and male smoking frequency (last 3 months).

Smoking Frequency	IUI (pregnancy)	IUI (no pregnancy)
Never	216	259
Rarely (1-4 times per month)	12	12
Sometimes (2-6 times per week)	4	2
Daily	2	9
Chi-square Test Indep. P-value	0.205	

Supplemental Table 7b. IUI pregnancy and female smoking frequency (last 3 months).

Smoking Frequency	IUI (pregnancy)	IUI (no pregnancy)
Never	227	268
Rarely (1-4 times per month)	3	5
Sometimes (2-6 times per week)	0	1
Daily	2	5
Chi-square Test Indep. P-value	0.599	

Supplemental Table 7c. IVF pregnancy and male smoking frequency (last 3 months).

Smoking Frequency	IVF (pregnancy)	IVF (no pregnancy)
Never	176	46
Rarely (1-4 times per month)	2	2
Sometimes (2-6 times per week)	0	0
Daily	2	2
Chi-square Test Indep. P-value	Too many missing values	

Supplemental Table 7d. IVF pregnancy and female smoking frequency (last 3 months).

Smoking Frequency	IVF (pregnancy)	IVF (no pregnancy)
Never	172	39
Rarely (1-4 times per month)	3	6
Sometimes (2-6 times per week)	3	0
Daily	1	1
Chi-square Test Indep. P-value	0.003	

Supplemental Table 8a. IUI live birth and male smoking frequency (last 3 months).

Smoking Frequency	IUI (live birth)	IUI (no live birth)
Never	156	319
Rarely (1-4 times per month)	8	16
Sometimes (2-6 times per week)	3	3
Daily	2	9
Chi-square Test Indep. P-value	0.599	

Supplemental Table 8b. IUI live birth and female smoking frequency (last 3 months).

Smoking Frequency	IUI (live birth)	IUI (no live birth)
Never	165	330
Rarely (1-4 times per month)	3	5
Sometimes (2-6 times per week)	0	1
Daily	2	5
Chi-square Test Indep. P-value	0.889	

Supplemental Table 8c. IVF live birth and male smoking frequency (last 3 months).

Smoking Frequency	IVF (live birth)	IVF (no live birth)
Never	136	86
Rarely (1-4 times per month)	2	2
Sometimes (2-6 times per week)	0	0
Daily	2	0
Chi-square Test Indep. P-value	Too many missing values	

Supplemental Table 8d. IVF live birth and female smoking frequency (last 3 months).

Smoking Frequency	IVF (live birth)	IVF (no live birth)
Never	131	80
Rarely (1-4 times per month)	3	6
Sometimes (2-6 times per week)	2	1
Daily	1	1
Chi-square Test Indep. P-value	0.371	

Supplemental Table 9a. IUI pregnancy and male alcohol consumption (last 3 months).

Smoking Frequency	IUI (pregnancy)	IUI (no pregnancy)
Never	123	179
Rarely (1-4 times per month)	65	61
Sometimes (2-6 times per week)	42	38
Daily	4	5
Chi-square Test Indep. P-value	0.101	

Supplemental Table 9b. IUI pregnancy and female alcohol consumption (last 3 months).

Smoking Frequency	IUI (pregnancy)	IUI (no pregnancy)
Never	146	189
Rarely (1-4 times per month)	64	66
Sometimes (2-6 times per week)	22	22
Daily	0	2
Chi-square Test Indep. P-value	0.354	

Supplemental Table 9c. IVF pregnancy and male alcohol consumption (last 3 months).

Smoking Frequency	IVF (pregnancy)	IVF (no pregnancy)
Never	80	23
Rarely (1-4 times per month)	49	13
Sometimes (2-6 times per week)	44	11
Daily	7	1
Chi-square Test Indep. P-value	0.921	

Supplemental Table 9d. IVF pregnancy and female alcohol consumption (last 3 months).

Smoking Frequency	IVF (pregnancy)	IVF (no pregnancy)
Never	87	24
Rarely (1-4 times per month)	58	13
Sometimes (2-6 times per week)	33	8
Daily	1	1
Chi-square Test Indep. P-value	0.708	

Supplemental Table 10a. IUI live birth and male alcohol consumption (last 3 months).

Smoking Frequency	IUI (live birth)	IUI (no live birth)
Never	89	213
Rarely (1-4 times per month)	49	77
Sometimes (2-6 times per week)	29	51
Daily	2	7
Chi-square Test Indep. P-value	0.209	

Supplemental Table 10b. IUI live birth and female alcohol consumption (last 3 months).

Smoking Frequency	IUI (live birth)	IUI (no live birth)
Never	110	225
Rarely (1-4 times per month)	43	87
Sometimes (2-6 times per week)	17	27
Daily	0	2
Chi-square Test Indep. P-value	0.660	

Supplemental Table 10c. IVF live birth and male alcohol consumption (last 3 months).

Smoking Frequency	IVF (live birth)	IVF (no live birth)
Never	58	45
Rarely (1-4 times per month)	39	23
Sometimes (2-6 times per week)	37	18
Daily	6	2
Chi-square Test Indep. P-value	0.456	

Supplemental Table 10d. IVF live birth and female alcohol consumption (last 3 months).

Smoking Frequency	IVF (live birth)	IVF (no live birth)
Never	61	50
Rarely (1-4 times per month)	48	23
Sometimes (2-6 times per week)	28	13
Daily	0	2
Chi-square Test Indep. P-value	0.071	

Supplemental Table 11a. IUI pregnancy and male physical activity (last 3 months).

Male Physical Activity	IUI (pregnancy)	IUI (no pregnancy)
Low	69	102
Moderate	83	102
High	81	77
Chi-square Test Indep. P-value	0.137	

Supplemental Table 11b. IVF pregnancy and male physical activity (last 3 months).

Male Physical Activity	IVF (pregnancy)	IVF (no pregnancy)
Low	73	15
Moderate	60	19
High	44	14
Chi-square Test Indep. P-value	0.453	

Supplemental Table 11c. IUI live birth and male physical activity (last 3 months).

Male Physical Activity	IUI (live birth)	IUI (no live birth)
Low	53	118
Moderate	55	130
High	60	98
Chi-square Test Indep. P-value	0.227	

Supplemental Table 11d. IVF live birth and male physical activity (last 3 months).

Male Physical Activity	IVF (live birth)	IVF (no live birth)
Low	56	32
Moderate	48	31
High	33	25
Chi-square Test Indep. P-value	0.716	
